# An association study of *FOXO3* variant and longevity

**DOI:** 10.1590/1678-4685-GMB-2017-0169

**Published:** 2018-06-11

**Authors:** Geralda Gillian Silva-Sena, Daniela Camporez, Lígia Ramos dos Santos, Aline Sesana da Silva, Lúcia Helena Sagrillo Pimassoni, Alessandra Tieppo, Maria do Carmo Pimentel Batitucci, Renato Lírio Morelato, Flavia de Paula

**Affiliations:** 1 Universidade Federal do Espírito Santo Universidade Federal do Espírito Santo Programa de Pós-Graduação em Biotecnologia VitóriaES Brazil Programa de Pós-Graduação em Biotecnologia, Renorbio, Universidade Federal do Espírito Santo, Vitória, ES, Brazil; 2 Universidade Federal do Espírito Santo Universidade Federal do Espírito Santo Departamento de Educação Integrada em Saúde VitóriaES Brazil Departamento de Educação Integrada em Saúde, Centro de Ciências da Saúde, Universidade Federal do Espírito Santo, Vitória, ES, Brazil; 3 Universidade Federal do Espírito Santo Universidade Federal do Espírito Santo Departamento de Ciências Biológicas VitóriaES Brazil Departamento de Ciências Biológicas, Centro de Ciências Humanas e Naturais, Universidade Federal do Espírito Santo, Vitória, ES, Brazil; 4 Escola Superior de Ciências da Santa Casa de Misericórdia de Vitória Escola Superior de Ciências da Santa Casa de Misericórdia de Vitória VitóriaES Brazil Escola Superior de Ciências da Santa Casa de Misericórdia de Vitória, Vitória, ES, Brazil; 5 Hospital da Santa Casa de Misericórdia de Vitória Hospital da Santa Casa de Misericórdia de Vitória VitóriaES Brazil Hospital da Santa Casa de Misericórdia de Vitória, Vitória, ES, Brazil; 6 Universidade Federal do Espírito Santo Universidade Federal do Espírito Santo Programa de Pós-Graduação em Ciências Farmacêuticas VitóriaES Brazil Programa de Pós-Graduação em Ciências Farmacêuticas, Centro de Ciências da Saúde, Universidade Federal do Espírito Santo, Vitória, ES, Brazil

**Keywords:** Lifespan, SNPs, environmental factors, oxidative stress, genomic damage

## Abstract

Human longevity is a polygenic and multifactorial trait. Pathways related to lifespan are complex and involve molecular, cellular, and environmental processes. In this analytical observational study, we evaluated the relationship between environment factors, oxidative stress status, DNA integrity level, and the association of *FOXO3* (rs2802292), *SOD2* (rs4880), *APOE* (rs429358 and rs7412), and *SIRT1* (rs2273773) polymorphisms with longevity in oldest-old individuals from southeastern Brazil. We found an association between the *FOXO3* GG genotype and gender. While lifestyle, anthropometric, and biochemical characteristics showed significant results, DNA damage and oxidative stress were not related to lifespan. We found that long-lived individuals with *FOXO3* GT genotype had low levels of triglycerides. This study is the first to demonstrate that *FOXO3* could be a candidate gene for longevity in the Brazilian population. These results are important in terms of provisions of health care for age-related diseases and lifespan, and provide insight for further research on epigenetic, gene regulation, and expression in oldest-old individuals.

## Introduction

Life expectancy in the world has more than doubled in the last two centuries. People aged 85 years or more, often designated the “oldest-old”, are the fastest-growing age group. Longevity is a multifactorial condition, affected by environmental and genetic factors, as well as by oxidative and genomic damage. Several hypotheses have been postulated to explain aging and lifespan, which have attracted widespread scientific and public interest (Brooks-Wilson, 2013; Simm and Klotz, 2015). The reactive oxygen species (ROS) theory of aging is related to oxidative stress and macromolecule damage. Twin-based research has shown that the genetic contribution is approximately 25% and becomes more profound after the age of 85 (Perls *et al.*, 2000). Single nucleotide polymorphisms (SNPs) are commonly used in human longevity studies to investigate common variants associated with lifespan.

The *Forkhead box O3* (*FOXO3*) gene mediates metabolic and oxidative stress, and participates in the insulin/insulin-like growth factor-1 signaling (IIS) pathway. Because of this, rs2802292 *FOXO3* has been associated with lifespan (Soerensen *et al.*, 2015). Also involved with longevity is the *SOD2* (*Superoxide Dismutase 2*) gene, which encodes a manganese-dependent superoxide dismutase enzyme (Mn-SOD) and is implicated with oxidative stress. Among the SNPs in *SOD2* related to longevity, rs4880 is the most studied (Gentschew *et al.*, 2013). Another gene largely studied in reference to longevity and diseases that affect older people has been the *Apolipoprotein E* gene (*APOE*). Two SNPs, rs429358 and rs7412, encode a protein of relevance in the process of lipid metabolism ([Bibr B53] Zhong *et al.*, 2016). *Sirtuin 1*, or *SIRT1*, (Silent Information Regulator Type 1) is a NAD^+^-dependent deacetylase that belongs to a family of SIR proteins. *SIRT1* rs2273773 has been shown to be involved in DNA repair, resistance to oxidative stress, and lifespan (Howitz *et al.*, 2003).

Studies on the association between longevity and genetic, oxidative, and genomic damage markers have significant clinical importance (Fragoso *et al.*, 2015). However, these works have often yielded conflicting results and not all genetic variants have been replicated. Therefore, the main objective of this work was to study the association of *FOXO3* (rs2802292), *SOD2* (rs4880), *APOE* (rs429358 and rs7412), and *SIRT1* (rs2273773) polymorphisms with longevity, oxidative stress status, and DNA integrity level in oldest-old individuals from southeastern Brazil.

## Materials and Methods

### Subjects

This is an observational and analytical study of 452 unrelated individuals. The sample of long-lived individuals (LLI) included 220 participants with age ≥85 years. The control group had 232 elders with ages between 70-75 years, which is close to the 73.5-year-average lifespan of the Brazilian population (Instituto Brasileiro de Geografia e Estatística, 2010). The chosen age range of controls is in accordance with studies, which claim that elderly people with age close to the lifespan of a certain population are more prone to genetic factors then to environmental factors (Willcox *et al.*, 2008; Anselmi *et al.*, 2009; Flachsbart *et al.*, 2009). Moreover, there is no data about mortality control in Brazil. Likewise, recent predictions by the Instituto Brasileiro de Geografia e Estatística (2013), in the Complete Mortality Table for Brazil, state that the Brazilian elderly population presented life expectancy from 84.7 years to the exact age of 70 years and from 86.7 years to the exact age of 75. Therefore, it is expected that a small portion of the controls admitted in our study will reach the age established for the LLI group, since they presented a mean survival time of 13.2 years (average between 70-84.7 and 75-86.7 years). In each group, sex and age were matched.

All the selected participants were from the metropolitan region of Espírito Santo, Grande Vitória, in Southeast Brazil. A geriatrician assisted all participants for 20 years in the Geriatric Unit of the Santa Casa de Misericórdia de Vitória Hospital resting home, (Abrigo à Velhice Desamparada Alta Loureiro Machado, ES, Brazil). This study was approved by the Committee of Human Research of the Universidade Federal do Espírito Santo, Health Sciences Center, Brazil, and performed in accordance with The Code of Ethics of the World Medical Association. To participate in the study, elders or their relatives gave written informed consent. Each individual answered a questionnaire adapted from the International Commission for Protection against Environmental Mutagens and Carcinogens (Carrano and Natarajan, 1988), the Program Gênesis-Gravataí (Flores *et al.*, 2013), and the Survey on Quality of Life Short Form 36 - SF36, adapted to the Brazilian population (Ware and Sherbourne, 1992). Questions were about demographic and socioeconomic characteristics, such as age, sex, ethnicity, education and income, smoking and alcohol consumption, physical activity, diet, and medical issues like vaccinations, medication, chronic diseases, mental health, and functional ability. A face-to-face interview with the participants was done by trained researchers. Medication and the presence of disease were confirmed through the patients’ records.

### Blood sampling

The blood samples were collected in 2014 and 2015. Eight milliliters of peripheral blood was collected into a 5% ethylene diamine tetraacetic acid (EDTA) tube (Vacuette, Greiner Labortechnik, Germany). For genotyping, samples were stored at 4 °C prior to analyses. For the oxidative stress and genomic damage analyses, 4 mL of blood were reserved. Genomic DNA was extracted according to previous methodology (Miller *et al.*, 1988). Concentration and purity of genomic DNA were measured using a NanoDrop 2000 Spectrophotometer (Thermo Fisher Scientific, Delaware, USA).

### Anthropometric, physical and biochemical data

Measurements of body mass index (BMI) and waist-hip ratio (W/H) were taken from the subjects. Height and weight were assessed with a stadiometer and a scale (Filizola, São Paulo, Brazil), and the hip and waist measurements were taken with a non-extendable anthropometric tape (Sanny, São Paulo, Brazil). Subjects were categorized into BMI groups according to Panamerican Health Organization (Organizacão Pan-Americana da Saúde, 2003) criteria. For the waist-hip ratio classification, cut-off point values were adopted: <1.0 for men and <0.85 for women (World Health Organization, 2000). Biochemical and physical data were defined using the specific criteria of Xavier *et al.* (2013) and Oliveira *et al.* (2015) and assessed through patients’ records.

### Oxidative stress analysis and comet assay

Participants selected for malondialdehyde (MDA) analysis, an oxidative stress parameter, and alkaline comet assay had to be non-smokers, not be taking antioxidant supplements, not consume alcohol, not have recently undergone x-ray scans, and not have recently undergone surgeries. A total of 100 participants, 50 LLI and 50 controls, were evaluated for MDA levels. Plasma samples of subjects were analyzed for MDA levels by high performance liquid chromatography with diode-array detection (HPLC-DAD) (Antunes *et al.*, 2008) and run in duplicate. Average values were reported as μmol/L of plasma.

To investigate genotoxic damage to peripheral blood cells, 15 LLI and 15 controls were evaluated using an alkaline comet assay, as previously described (Singh *et al.*, 1988; Tice *et al.*, 2000). DNA damage was determined by analysis of 100 randomly selected cells from each individual, and measurement of tail size, scored visually, was divided into four categories, ranging from no tail (no damage) to maximally long tails (maximum damage).

### Genotyping

*FOXO3* (rs2802292:G>T, RefSeqNM_001455.3), *SOD2* (rs4880:T>C, RefSeqNG_008729.1), *SIRT1* (rs2273773:T>C, RefSeqNM_001142498.1), and *APOE* (rs429358:T>C, RefSeqNG_007084.2 and rs7412:C>T, RefSeqNG_007084.2) SNPs were genotyped for 449 individuals by real-time polymerase chain reaction (qPCR). Thirty ηg/μL of genomic DNA were used for qPCR according to the manufacturer’s instructions (TaqMan SNP Genotyping Assay - Applied Biosystems, Carlsbad, California, USA), and the reactions were performed on a Rotor-Gene Q (Qiagen, Hilden, Germany). Genotypes were analyzed using the Rotor Gene Q Series Software v.2.1 (Qiagen). To validate the standard genotypes found in each SNP, Sanger sequencing was performed on an ABI PRISM 3130XL Genetic Analyzer/HITACHI (Applied Biosystems). The sequencing analysis was performed using the BioEdit software v.7.2.5 for windows (Ibis Biosciences, Carlsbad, California, USA).

### Statistical analysis

Statistical analysis of the data was performed using SPSS software v 23.0 for Windows (IBM corporation, Armonk, New York, USA) and *p*<0.05 values were considered significant. To test the association between longevity and the rs2802292 *FOXO3*, rs4880 *SOD2,* rs429358 and rs7412 *APOE*, and rs2273773 *SIRT1* polymorphisms, Pearson’s Chi-square or Fisher’s exact tests, with odds ratio (OR) and confidence intervals (CI) of 95%, were carried out. Moreover, the Hardy-Weinberg Equilibrium (HWE) was calculated *(p*<0.05).

Frequencies of demographic and socioeconomic characteristics were compared for each gender and for both groups (controls and long-lived) using Pearson’s Chi-square or Fisher’s exact tests for categorical variables, and Student’s *t*-tests for continuous variables. For the comparison of biochemical, physical, anthropometric characteristics, and oxidative stress status between LLI and control groups, Student’s t-test was used for continuous variables. The normality of the data was verified. For genomic damage analysis, the comparison between LLI and controls was performed using a Mann-Whitney test.

To evaluate the distribution of the oxidative damage product, DNA damage, clinical, anthropometric, and biochemical characteristics according to *FOXO3* genotypes, within long-lived individuals and controls, we used one-way analysis of variance (ANOVA), followed by Tukey and Pearson’s Chi-square tests for categorical variables. To test the association between *FOXO3, SOD2, APOE,* and *SIRT1* genotypes and the health status of long-lived individuals, Pearson’s Chi-square or Fisher’s exact tests were used. “Healthy” was defined as absence of chronic diseases (cardiovascular disease, diabetes, cancer, neurodegenerative, and respiratory diseases) and good functional ability (Willcox *et al.*, 2008; Ware and Sherbourne, 1992).

## Results

Demographic, socioeconomic, anthropometric, biochemical, and physical characteristics of the groups as well as oxidative stress status and genomic damage, are shown in Table 1. The average ages are 72.4 ± 1.7 years for controls and 89.3 ± 4.6 years for LLI. Female participants are predominant in the sample, as well as Caucasian individuals. Most individuals live in their own homes. Among the long-lived individuals, 63.2% were 85–89 years old, 33.6% were nonagenarians, and 3.2% were centenarians.

**Table 1:** Characteristics of study sample.

Characteristics (n)	LLI			Controls			*p* **
Gender (452)	Men (64)	Women (156)	p*	Men (75)	Women (157)	p*	
Age (452)	88.8 ± 4.1	89.3 ± 4.6	0.467	72.3 ± 1.8	72.4 ± 1.7	0.457	0.000
Ethnicity (449)							
Caucasian	38 (17.4%)	93 (42.5%)		48 (20.9%)	100 (43.5%)		
Black	9 (4.1%)	26 (11.9%)	0.898	12 (5.2%)	21 (9.1%)	0.755	0.612
Brown	16 (7.3%)	37 (16.9%)		14 (6.1%)	35 (15.2%)		
Descent Family - Italy (87)	37 (42.5%)		50 (57.5%)				0.099
Marital status (330)							0.000
Married	42 (25.8%)		85 (50.9%)				
Widower	94 (57.6%)		53 (31.7%)				
Separated/divorced/never married	27 (16.6%)		29 (17.4%)				
Monthly Income ($200.00) (297)	150 (50.5%)		147 (49.5%)				
Years of Education (325)							0.015
0 years	15 (9.9%)	51 (31.7%)	0.255	9 (5.5%)	34 (20.7%)		
1-4 years	29 (18.0%)	48 (29.8%)		34 (20.7%)	61 (37.2%)	0.325	
5-8 years	4 (2.5%)	11 (6.8%)		8 (4.9%)	14 (8.5%)		
> 8 years	1 (0.6%)	1 (0.6%)		1 (0.6%)	3 (1.8%)		
Home (290)							0.108
Own home	119 (88.1%)			145 (93.5%)			
Rented/ Live with other	16 (11.9%)			10 (6.5%)			
Biochemical physical and anthropometric data							
Diastolic Blood Pressure (mmHg) (299)	77.4 ± 10.4			79.5 ± 11.3			0.675
Systolic Blood Pressure (mmHg) (299)	133.9 ± 20.1			134.9 ± 20.7			0.093
Cholesterol (mg/dl) (257)	181.9 ± 39.9			187.9 ± 43.6			0.256
High-Density Lipoprotein Cholesterol (mg/dl) (246)	48.8 ± 10.6			46.7 ± 12.8			0.177
Low-Density Lipoprotein Cholesterol (mg/dl) (242)	110.1 ± 33.6			111.9 ± 38.7			0.701
Triglycerides (mg/dl) (249)	120.9 ± 73.6			152.0 ± 111.3			0.009
Glucose (mg/dl) (279)	106.9 ± 28.3			118.1 ± 47.4			0.017
Waist:hip ratio (265)	0.9 ± 0.1			0.9 ± 0.1			0.984
BMI (251)							0.003
< 23 kg/m^2^ (Underweight)	36 (31.3%)			22 (16.2%)			
23 |- 28 kg/m^2^ (Normal weight)	58 (50.4%)			64 (47.1%)			
28 |-30 kg/m^2^(Overweight)	7 (6.1%)			18 (13.2%)			
> = 30 kg/m^2^ (Obesity)	14 (12.2%)			32 (23.5%)			
Eat Vegetables (5 times/day) (309)							0.174
Yes	122 (39.5%)			128 (41.4%)			
No	23 (7.4%)			36 (11.7%)			
Eat Fruits (5 times/day) (309)							0.052
Yes	124 (40.1%)			126 (40.8%)			
No	21 (6.8%)			38 (12.3%)			
Alcohol consumption (326)							0.019
Use	6 (3.8%)	4 (2.5%)	0.071	18 (10.7%)	6 (3.6%)	0.000	
No use	43 (27.2%)	105 (66.5%)		36 (21.4%)	108 (64.3%)		
Smoker Status (328)							0.229
Never smoked	21 (13.3%)	73 (46.2%)	0.008	21 (12.4%)	95 (55.9%)		
Former smoked	25 (15.8%)	30 (19.0%)		30 (17.6%)	18 (10.6%)	0.000	
Current smoked	4 (2.5%)	5 (3.2%)		5 (2.9%)	1 (0.6%)		
Physical activity (283)							0.004
Yes	11 (24.4%)	10 (11.2%)	0.047	17 (38.6%)	28 (26.7%)	0.147	
No	34 (75.6%)	79 (88.8%)		27 (61.4%)	77 (73.3%)		
Family history (68)							0.006
Cardiovascular Disease	13 (44.8%)		4 (10.5%)				
Diabetes	5 (17.2%)		11(28.9%)				
Cancer	11 (38.0%)		23 (60.5%)				
Malondialdehyde (μmol/L plasma) (100)	1.99 ± 0.66	2.03 ± 0.73	0.879	1.91 ± 0.62	1.77 ± 0.63	0.447	0.137
Genomic damage (a.u.) (30)^b^	0.044 ± 0.011		0.031 ± 0.007				0.567

The data are presented as mean ± SD or SEM

b (standard deviation or standard error of the mean) or number of subjects with percentage in parentheses. Abbreviations: n - number of individuals; LLI - Long-Lived Individuals; a.u. - arbitrary units;

* Comparison between gender;

** Comparison between Controls and LLI; *p*-values are uncorrected; the adjusted residue of χ2 test were used for significant data.

A significant difference in marital status was observed for married controls (50.9%) and LLI widowers (57.6%) (*p*=0.000). Most participants had one to four years of formal education, with a significant difference between groups (*p*=0.015); the LLI group had a higher number of individuals who lacked formal education. They also showed lower triglycerides (*p*=0.009), lower glucose (*p*=0.017), and low BMI (*p*=0.003). We observed that the LLI had a higher fruit intake per day, although this had a borderline significance (*p*=0.052). In the sample as a whole, the average BMI was 26.00 ± 4.57 kg/m^2^. According to the W:H ratio, 19.4% of men and 45.4% of women presented risk for metabolic disease (data not shown).

Most individuals did not consume alcohol. We observed a significant difference in alcohol consumption between groups (*p*=0.019). Men and women in the control sample drank less alcohol (*p*=0.000).

Concerning smoking, we found a higher proportion of women who never smoked, of men who quit smoking, and of men who currently smoke, both in long-lived (*p*=0.008) and control (*p*=0.000) groups.

Most individuals reported they did not exercise. Comparing the groups, there was a difference in the proportion of people who did not exercise (*p*=0.004). In LLI, the difference in proportion between men and women was slightly significant (*p*=0.047).

As for medical family history, cancer was more frequently observed in controls (60.5%), whereas heart disease was more frequently observed in LLI (44.8%). The difference in proportion between controls and LLI was significant (p=0.006). In both malondialdehyde and DNA damage analyses, average levels were higher in LLI than in controls, although non-significant.

The genotype and allele frequencies of *FOXO3 (*rs2802292), *SOD2 (*rs4880), *APOE* (rs429358 and rs7412), and *SIRT1 (*rs2273773) for both genders, LLI, and controls are shown in Table 2. In the LLI group, an association was observed between *FOXO3* GG genotype and gender (OR=0.348; 95% CI=0.139-0.873; *p*=0.02). However, no association was found between this genotype and longevity, considering the stratification of the sample in elderly men (OR=0.414; 95% CI=0.150-1.142; *p*=0.088) and elderly women (OR=1.137; 95% CI=0.666-1.941; *p*=0,639) within the two groups. No significant difference was observed for the other SNPs. All LLI and control polymorphisms were found in HWE.

**Table 2:** Distribution of genotypes and alleles in study groups.

	Groups (n)	LLI (218)					Controls (231)					OR(95% CI)**	*p* **	*p* ***
Genes	Genotypes	Men	Women	Total	OR (95% CI)*	*p* *	Men	Women	Total	OR (95% CI)*	*p* *			
		n (%)	n (%)	n (%)			n (%)	n (%)	n (%)					
					0.348					0.955		0.910		
*FOXO3*	GG	6 (2.8)	36 (16.5)	42 (19.3)	(0.139-0.873)	0.020	15 (6.5)	33 (14.3)	48 (20.8)	(0.482-1.894)	0.896	(0.573-1.445)	0.689	0.907
					1.425					0.869		1.073		
	GT	38(17.4)	80 (36.7)	118(54.1)	(0.786-2.583)	0.242	37 (16.0)	84 (36.4)	121(52.4)	(0.500-1.511)	0.619	(0.740-1.555)	0.711	
					1.284					1.238		0.988		
	TT	19 (8.7)	39 (17.9)	58 (26.6)	(0.671-2.458)	0.449	22 (9.5)	40 (17.3)	62 (26.8)	(0.670 - 2.287)	0.496	(0.650-1.501)	0.955	
*SOD2*														
					0.841					1.412		0.987		
	CC	15 (6.9)	42 (19.3)	57 (26.1)	(0.426-1.659)	0.617	23 (10.0)	38 (16.5)	61 (26.4)	(0.765-2.607)	0.269	(0.648-1.502)	0.950	0.369
					1.070					0.821		1.238		
	CT	36(16.5)	86 (39.4)	122(56.0)	(0.592-1.932)	0.823	35 (15.1)	82 (35.5)	117(50.6)	(0.472-1.428)	0.484	(0.854-1.795)	0.259	
					1.115					0.895		0.732		
	TT	12 (5.5)	27 (12.4)	39 (17.9)	(0.525-2.370)	0.776	16(6.9)	37 (16.0)	53 (22.9)	(0.460-1.740)	0.743	(0.461-1.162)	0.185	
*APOE*														
					1.291					0.996		1.088		
	∊2∊3	10 (4.7)	16 (7.5)	26 (12.2)	(0.553-3.012)	0.658	8 (3.8)	20 (9.4)	28 (13.2)	(0.412-2.401)	1.000	(0.615-1.927)	0.884	0.973
					1.493					0.904		1.079		
	∊3∊3	43(20.1)	72 (33.8)	115(53.9)	(0.837-2.662)	0.191	33 (15.5)	86 (40.4)	119(55.9)	(0.498-1.643)	0.762	(0.736-1.580)	0.770	
					0.492					2.542		0.796		
	∊2∊4	1 (0.5)	4 (1.9)	5 (2.4)	(0.054-1.889)	0.667	2 (0.9)	2 (0.9)	4 (1.8)	(0.350-18.468)	0.324	(0.211-3.006)	1.000	
					0.547					1.105		0.885		
	∊3∊4	14 (6.6)	44 (20.7)	58 (27.3)	(0.276-1.084)	0.102	16 (7.5)	37 (17.4)	53 (24.9)	(0.560-2.182)	0.861	(0.574-1.365)	0.659	
					1.000					0.702		1.000		
	∊4∊4	3(1.4)	6 (2.8)	9 (4.2)	(0.243-4.121)	1.000	2 (0.9)	7 (3.3)	9 (4.2)	(0.142-3.479)	1.000	(0.389-2.570)	1.000	
*SIRT1*														
					2.508					2.137		2.140		
	CC	2 (0.9)	2 (0.9)	4 (1.8)	(0.346-18.208)	0.347	1 (0.4)	1 (0.4)	2 (0.8)	(0.132-34.643)	0.584	(0.388-11.804)	0.438	0.660
					0.936					0.661		0.945		
	CT	10 (4.6)	26 (11.9)	36 (16.5)	(0.422-2.076)	0.871	10 (4.3)	30 (13.0)	40 (17.3)	(0.304-1.437)	0.294	(0.576-1.548)	0.821	
					0.937					1.409		0.989		
	TT	51(23.4)	127(58.3)	178(81.7)	(0.442-1.984)	0.865	63 (27.3)	126(54.5)	189(81.8)	(0.665-2.987)	0.370	(0.613-1.596)	0.964	
		LLI	Controls											
	Allele	*n*	*%*	*n*	*%*	*p* **	OR (95%CI)							
							0.975							
*FOXO3*	G	202	46.3	217	47.0	0.848	(0.750-1.267)							
							1.026							
	T	234	53.7	245	53.0	0.472	(0.789-1.334)							
*SOD2*	C	236	54.1	239	51.7		1.101							
							(0.847-1.431)							
	T	200	45.9	223	48.3		0.908							
							(0.699-1.181)							
*APOE*	∊2	31	7.3	32	7.5	0.867	0.904							
							(0.542-1.509)							
	∊3	314	73.7	319	74.9		0.768							
							(0.576-1.024)							
	∊4	81	19.0	75	17.6		1.018							
							(0.721-1.438)							
*SIRT1*	C	44	10.1	44	9.5	0.775	1.066							
							(0.687-1.656)							
							0.938							
	T	392	89.9	418	90.5		(0.604-1.456)							

Abbreviations: n= number of individuals; LLI = Long-Lived Individuals; OR = Odds Ratio; CI = Confidence Interval;

* Comparison between gender;

** Comparison between CT and LLI;

*** *p*-values are uncorrected; For *APOE*, n= 213 (LLI) and n= 213 (Controls). *FOXO3* (rs2802292:G>T. RefSeqNM_001455.3), *SOD2* (rs4880:T>C. RefSeqNG_008729.1*), SIRT1* (rs2273773:T>C. RefSeqNM_001142498.1) and *APOE* (rs429358:T>C. RefSeqNG_007084.2 and rs7412:C>T. RefSeqNG_007084.2).

Comparing the distribution of biochemical variables with the *FOXO3* genotypes, there was significant difference between the average triglyceride levels among LLI (*p*=0.036): individuals with the TG genotype showed low levels of triglycerides, while individuals with the TT genotype showed high levels of triglycerides. For other variables (clinical variables, and oxidative and genomic damage) no significant difference was found (Table 3). The distribution of *FOXO3, SOD2, APOE,* and *SIRT1* genotypes between healthy and frail LLI was also assessed, and data are shown in Table 4, though no significant difference was observed. We did not find an association of protective genotypes between health status and longevity.

**Table 3:** Biochemical, anthropometric, clinical variables, oxidative and genomic damages according *FOXO3* genotypes.

	*FOXO3* SNP Genotypes (rs2802292)			
Variables (n)/Groups	GG	GT	TT	*p* *
Triglycerides (mg/dl) (249)				
LLI	108.2 ± 34.0	109.8 ± 63.6^a^	147.6 ± 96.9^a^	0.036
Controls	150.1 ± 131.6	148.6 ± 83.7	163.1 ± 140.2	0.818
Glucose (mg/dl) (279)				
LLI	109.2 ± 37.5	106.1 ± 26.4	107.1 ± 25.1	0.896
Controls	122.8 ± 50.6	119.6 ± 48.9	106.8 ± 25.7	0.226
Body Mass Index (251)				
LLI	25.1 ± 3.7	24.3 ± 4.7	26.7 ± 4.7	0.070
Controls	26.7 ± 4.4	26.8 ± 4.6	27.4 ± 4.8	0.760
Heart disease (236)				
LLI	19 (45.2%)	65 (55.1%)	30 (51.7%)	0.545**
Controls	26 (54.2%)	61 (50.4%)	35 (56.5%)	0.725**
Diabetes (108)				
LLI	6 (14.3%)	24 (20.3%)	10 (17.2%)	0.663**
Controls	14 (29.2%)	36 (29.8%)	18 (29.0%)	0.994**
Malondialdehyde (μmol/L plasma) (100)				
LLI	1.68 ± 0.23	1.91 ± 0.66	2.28 ± 0.74	0.116
Controls	1.88 ± 0.75	1.81 ± 0.55	1.58 ± 0.24	0.393
Genomic damage (a.u.) (30)^b^				
LLI	0.015 ± 0.009	0.065 ± 0.017	0.027 ± 0.012	0.108
Controls	0.045 ± 0.025	0.027 ± 0.008	0.032 ± 0.014	0.731

The data are presented as mean ± SD or SEM

b (standard deviation or standard error of the mean) or number of subjects with percentage in parentheses. Abbreviations: n - number of individuals; LLI - Long-Lived Individuals; a.u. - arbitrary units;

* Comparison between mean values of parameters for genotypes within LLI and controls;

** Odds Ratio was not calculated because *p* > 0.05;

a *p* < 0.05 (Tukey test); For heart disease and diabetes, values refer to that morbidity carrier.*FOXO3* (rs2802292:G > T. RefSeqNM_001455.3).

**Table 4:** Distribution of *FOXO3, SOD2, APOE and SIRT1* genotypes between healthy and frailty status of the long-lived individuals.

Gene	Genotypes/Groups (n)	Healthy LLI (83)	Frail LLI (135)	*p* *	OR (95% CI)**	*p* **
		n (%)	n (%)			
*FOXO3*	GG	19 (22.9)	23 (17)		1.446 (0.732 - 2.856)	0.287
	GT	43 (51.8)	75 (55.6)	0.567	0.860 (0.497 - 1.488)	0.590
	TT	21(25.3)	37 (27.4)		0.897 (0.482 - 1.673)	0.733
*SOD2*	CC	25 (30.1)	32 (23.7)		1.387 (0.751-2.565)	0.295
	CT	41(49.4)	81 (60.0)	0.342	0.651 (0.375-1.129)	0.125
	TT	17 (20.5)	22 (16.3)		1.323 (0.656-2.670)	0.433
*APOE*	∊2∊3	11 (13.6)	17 (12.9)		1.063 (0.471-2.400)	0.883
	∊3∊3	39 (48.1)	80 (60.6)		0.640 (0.345-1.055)	0.075
	∊2∊4	1 (1.2)	3 (2.3)	0.313	0.538 (0.055-5.257)	1.000
	∊3∊4	25 (30.9)	28 (21.2)		1.658 (0.883-3.112)	0.114
	∊4∊4	5 (6.2)	4 (3.0)		2.105 (0.548-8.080)	0.306
*SIRT1*	CC	3 (3.6)	1 (0.7)		5.025 (0.514-49.160)	0.156
	CT	12 (14.5)	24 (17.8)	0.292	0.782 (0.367-1.662)	0.577
	TT	68 (81.9)	110 (81.5)		1.030 (0.507-2.092)	1.000

Abbreviations: n - number of individuals; LLI - Long-Lived Individuals; OR - Odds Ratio; CI - Confidence Interval;

* Comparison between genotypes of frailty and healthy status;

** *p*-value for Odds Ratio; For *APOE*, n= 81 (Healthy LLI) and n= 132 (Frailty LLI*). FOXO3* (rs2802292:G > T. RefSeqNM_001455.3), *SOD2* (rs4880:T > C. RefSeqNG_008729.1), *SIRT1* (rs2273773:T > C. RefSeqNM_001142498.1) *APOE* (rs429358:T > C. RefSeqNG_007084.2 and rs7412:C > T. RefSeqNG_007084.2).

## Discussion

The present study aimed to evaluate the association of *FOXO3* (rs2802292), *SOD2* (rs4880), *APOE* (rs429358 and rs7412), and *SIRT1* (rs2273773) polymorphisms with longevity and the relationship between genomic damage and oxidative stress status in elderly people of southeastern Brazil. A polymorphism of *FOXO3* had an association with gender, and anthropometric and biochemical characteristics showed significant results. No relationship was found between longevity and DNA damage status or oxidative stress.

Environmental factors may play a role in age-related diseases and longevity, but the relative importance of these factors remains unclear. Previous studies have demonstrated that lifestyle, diet, socioeconomic, biochemical, and anthropometric characteristics affect the development of age-related diseases as well as the health and lifespan of the general population (Praticò, 2002; Britton *et al.*, 2008). Dutta *et al.* (2011) found no relationship between years of education and lifespan. In our study, we noted that 86.8% of controls and LLI had up to four years of education. We believe that this is because in the 1930s, these individuals had less access to education in Brazil. In addition, the typical monthly income of an elderly in our sample was $200.00, which could also explain the lower education levels. A majority of the LLI are widowed and of the controls, married. However, the Dutta *et al.* (2011) study, that accompanied elderly from 65 to 85 years, showed that on average, marital status did not influence the survival of participants.

Analysis of other biological factors in our study showed low levels of triglycerides and glucose, low BMI, low alcohol consumption (93.7%), and a tendency, in the LLI group, to eat more fruits per day. Dutta *et al.* (2011) had also shown that participants with a BMI of 30.0 kg/m^2^ were less likely to achieve longevity. In a longitudinal study, Hodge *et al.* (2014) noted that longevity was correlated with both fruit consumption and moderate alcohol consumption. In the Multinational MEDIS Study, the oldest-old had lower BMI levels and a prevalence of dyslipidemia, but no difference between controls and oldest-old relative to education status and marital status (Tyrovolas *et al.*, 2016). We believe these parameters are related with successful aging in our study.

DNA damage and products of oxidative stress have also been studied in relation to longevity. No difference in the levels of these two biomarkers was observed in our sample. Lower levels of malondialdehyde may not have been found because most of the studied individuals, in both control and LLI groups, had diseases related to aging that could promote oxidative imbalance. As for DNA integrity, studies show that ≥85-year-old individuals have levels of genomic lesions either higher than or similar to younger elders (Franzke *et al.*, 2015). However, our results characterize the frailty of aging *per se*, as discussed in Taufer *et al.* (2005). Moreover, we cannot rule out other defense pathways against oxygen reactive species and/or biomarkers that were not studied in the present work and which may affect longevity (Praticò, 2002; Saeed *et al.*, 2005). The hypothesis of oxidative stress resistance states that the increased genomic damage with age is accompanied by efficient antioxidant and repair mechanisms for successful aging (Franzke *et al.*, 2015).

In relation to candidate genes for longevity, Genome Wide Association Studies (GWAS) show that *APOE* and *FOXO3* are associated to human lifespan (Christensen *et al.*, 2006; Broer *et al.*, 2015). *SOD2* and *SIRT1* are also great predisposition genes, having been the focus of many studies (Taufer *et al.*, 2005; Flachsbart *et al.*, 2006; Soerensen *et al.*, 2009; Gentschew *et al.*, 2013; Han *et al.*, 2015).

*FOXO3* is localized in Ch6q21 and belongs to a subfamily of transcription factors that target longevity regulators, implicated in the insulin and insulin-like growth factor signaling pathways (Martins *et al.*, 2016). The FOXO3 protein is involved in diverse cellular and physiological processes, including cell proliferation, apoptosis, cellular responses to oxidative stress, cancer, cell cycle regulation, metabolism, and longevity (Tzivion *et al.*, 2011). The rs2802292 in *FOXO3* is a G > T change. A study of the Danish population investigated 15 SNPs in the *FOXO3* gene and involved 1088 participants (Soerensen *et al.*, 2015). This research showed a positive association between *FOXO3* rs2802292 and 4 other SNPs of this gene with phenotypes shown to predict survival in a combined sample of male and female oldest-old individuals. No association between *FOXO3* and type 2 diabetes was found in an elderly Indian population study, which included a sample of 994 type 2 diabetic individuals and 984 normoglycemic controls (Nair *et al.*, 2012). In our sample, we found a significant gender-related difference for GG genotype in the LLI group, although lifespan was not associated with the *FOXO3* GG genotype*,* in neither men nor women.

Unlike our study, Willcox *et al.* (2008) and Anselmi *et al.* (2009) found that the *FOXO3* GG genotype was associated with longevity in long-lived Japanese and Italian men, respectively. Willcox *et al.* (2008) also observed that the G allele and GG genotype frequencies tended to be increased in long-lived *vs* control men. A possible reason for the *FOXO3* SNP results found in our study is the small sample size (in gender-specific effect), which may decrease the statistical power to detect associations. *FOXO3* may play a role in determining longevity, probably by enabling those who have the protective genotype to be shielded in some way from oxidative stress, cell death, and glucose metabolism (Soerensen *et al.*, 2015). Moreover, in our work the oldest individuals carrying the G allele (in the GT genotype) of *FOXO3* had lower levels of triglycerides compared to individuals who were homozygous for the T allele. These results corroborated with Willcox *et al.*‘s (2008) work, which found that lower levels of triglyceride may be a phenotype related to healthy aging, and that individuals with at least one G allele have a higher protection factor for longevity compared to individuals homozygous for the T allele. Lower triglyceride values (≤150 mg/dL), similar to other variables, are inversely correlated with the increase of visceral adipose tissue and thus with a lower risk for metabolite disease development (Xavier *et al.*, 2013) that may culminate in an unsuccessful aging process. However, we did not identify an association between the G allele of *FOXO3* and lifespan in our work.

The SOD2 protein (Mn-SOD) is involved in oxidative stress regulation, which is a pathway that leads to longevity. It is a great defense against ROS in the mitochondria and acts at the matrix, converting superoxide radicals into hydrogen peroxide (da Cruz, 2015). The rs4880 in *SOD2* (Ch6q25.3) is a T > C that replaces valine for alanine, which may disturb the SOD2 protein activity and unbalance the oxidant-antioxidant equilibrium in the mitochondria (Shimoda-Matsubayashi *et al.*, 1996). Although this rs4880 SNP is the most studied in *SOD2* (Gentschew *et al.*, 2013), results from different studies are inconsistent. One of these works tested the association of the rs4880 SNP with longevity in a sample of 1650 long-lived individuals from Denmark, and observed that individuals with the C allele had decreased mortality (*p*=0.002) (Soerensen *et al.*, 2009). A study conducted in the south of Brazil tested for age-related mortality with 489 volunteers divided into three groups (newborns, 21-79-year-old adults, and 80-105-year-old elders), but no association was found (Taufer *et al.*, 2005). Gentschew *et al.* (2013) found no association in a study of 1612 long-lived individuals (> 95 years old) and 1104 controls (60-75 years old) in a German population. Similarly, we demonstrated that *SOD2* is not associated with human longevity in our population.

*APOE*, located on chromosome 19q13.2, has three different isoforms: ∊2 (cys112, cys158), ∊3 (cys112, arg158), and ∊4 (arg112, arg158), designated by two SNPs, rs429358 (T>C) and rs7412 (C>T). Because of its involvement in cholesterol transport processes, this protein can influence the route of lipids and can lead to oxidative stress, neuronal damage, and inflammation (Huebbe *et al.*, 2011). No association was found between *APOE* and hypertension in a population of 1406 elderly individuals from Bambuí, Brazil (Fuzikawa *et al.*, 2008). However, the ∊4 allele proved to be a risk factor for premature death in a GWAS study of a Canadian population of healthy oldest-old individuals (Tindale *et al.*, 2014). We did not see a link between *APOE* and longevity in our population.

*SIRT1* is a candidate gene for longevity and promoting health, located on chromosome 10q21.3. The SIRT1 protein resides in a nuclear compartment and is a member of a class I family of seven proteins. The activity of this protein depends on the NAD^+^/NADH ratio, a key indicator for oxygen consumption, suggesting that this protein has a physiological role in regulating metabolic homeostasis (Giblin *et al.*, 2014). Because of *SIRT1*’s potential role as a mediator of lifespan, *SIRT1* polymorphic variants, such as the rs2273773 T>C SNP, have been previously studied (Flachsbart *et al.*, 2006). However, only a small number of *SIRT1* SNP studies are related to lifespan in humans. During the last years, these polymorphic variants have been investigated in a context of metabolism or calorie restriction, and have been associated with aging disease-related phenotypes (Nogueiras *et al.*, 2012). This association is also supported by a study that showed that *SIRT1* genetic variation affects lipid profiles in a sample of 382 Ashkenazi Jews (Han *et al.*, 2015). Flachsbart *et al.* (2006) found no association for this SNP in a sample of 1245 long-lived German individuals. Comparably, our findings demonstrated no association between *SIRT1* variants and longevity in older individuals.

The present work shows a lack of association between *FOXO3* (rs2802292), *SOD2* (rs4880), *SIRT1* (rs2273773), and *APOE* (rs429358 and rs7412) with longevity. A possible reason for this result may be the small size of our sample. Longevity association studies frequently use large samples of around 1000 individuals for instance (Di Bona *et al.*, 2014; Broer *et al.*, 2015). However, this is the first longevity study in the state of Espírito Santo, Brazil, and we aim to expand the sample population. Another potential reason may be the different age ranges of the individuals in LLI and control groups. Some studies that have found an association with longevity used different age ranges for LLI and controls (Kilic *et al.*, 2015). Additionally, ethnicity may mask certain genetic marks for longevity, considering Brazilian populations are a mixture of Iberian Caucasians, West Africans, and Native Americans (Pena *et al.*, 2011). Each population has its own ethnic features, and allele and genotype frequencies can vary between different regions.

No relationship was observed between healthy oldest-old individuals and *FOXO3, SOD2, APOE*, and *SIRT1* genotype frequencies. This result can be explained in the context of the data from the Global Burden of Disease Study 2013 (Murray *et al.*, 2015), which showed that the healthy life expectancy of Brazilians, considering disability-adjusted life-years (DALYs) and healthy life expectancy (HALE), is 65 years. Among the LLI of our sample, 61.9% (135) had chronic-degenerative diseases and functional disabilities. Taking into consideration that all individuals in our study were at least 85 years old, our results corroborate with this information, as they were 20 years old or more beyond the healthy life expectative and thus, at risk for morbidities and disability.

In conclusion, although longevity is the result of multiple and complex features, our work suggests that environmental factors and *FOXO3* could have an intricate effect on human longevity. Our research contributes to the characterization of the complex mechanisms of aging and lifespan. It may also support the development of better treatments and offer the opportunity for diagnosis and prevention of age-related diseases, thus, postponing aging and/or prolonging healthy lifespan, and establishing more effective public health strategies. Other approaches, such as epigenetic control and gene regulation and expression, also warrant investigation because they can help to understand the mechanisms that regulate lifespan (Kilic *et al.*, 2015; Benayoun *et al.*, 2015). Overall, we believe that our study help to pave the way to a promising future of genomic geriatrics and personalized medicine.
